# Morvan’s syndrome and myasthenia gravis related to familial Mediterranean fever gene mutations

**DOI:** 10.1186/s12974-016-0533-7

**Published:** 2016-03-29

**Authors:** Junpei Koge, Shintaro Hayashi, Hiroyuki Murai, Jun Yokoyama, Yuri Mizuno, Taira Uehara, Naoyasu Ueda, Osamu Watanabe, Hiroshi Takashima, Jun-ichi Kira

**Affiliations:** Department of Neurology, Neurological Institute, Graduate School of Medical Sciences, Kyushu University, 3-1-1 Maidashi, Higashi-ku, Fukuoka 812-8582 Japan; Department of Neurological Therapeutics, Neurological Institute, Graduate School of Medical Sciences, Kyushu University, 3-1-1 Maidashi, Higashi-ku, Fukuoka 812-8582 Japan; Department of Medicine and Biosystemic Science, Kyushu University Faculty of Medicine, 3-1-1 Maidashi, Higashi-ku, Fukuoka 812-8582 Japan; Department of Neurology and Geriatrics, Kagoshima University Graduate School of Medical and Dental Sciences, 8-35-1 Sakuragaoka, Kagoshima, 890-8520 Japan

**Keywords:** Morvan’s syndrome, Myasthenia gravis, Anti-voltage-gated potassium channel complex antibodies, Myokymia, Familial Mediterranean fever

## Abstract

**Background:**

We present the first case of Morvan’s syndrome (MoS) and myasthenia gravis (MG) related to familial Mediterranean fever (FMF) gene mutations.

**Case presentation:**

A 40-year-old woman with a 1-year history of bilateral ptosis and limb muscle weakness presented to our hospital. She also had memory impairment, insomnia, hyperhidrosis, and muscle twitches. Electromyography confirmed widespread myokymia, and there was evidence of temporal region dysfunction on electroencephalography. Anti-voltage-gated potassium channel complex antibodies and anti-acetylcholine receptor antibodies were both positive. Edrophonium administration was effective for bilateral ptosis and muscle weakness. She and her family experienced self-limiting febrile attacks with arthralgia, which led us to suspect FMF. Genetic analyses revealed compound heterozygous mutations in exon 2 of the *MEFV* gene (L110P/E148Q). From these findings, a diagnosis of MoS and MG complicated with *MEFV* gene mutations was made. Intravenous high-dose corticosteroids, plasma exchange, and intravenous immunoglobulin resulted in only transient, limited improvement, and frequent relapses, especially in the myasthenic symptoms. Interleukin (IL)-6, IL-1β, and tumor necrosis factor-α were markedly elevated in the serum, which was considered to be derived from the *MEFV* mutations and responsible for the resistance to immunotherapy.

**Conclusion:**

The present case illustrates a possible link between auto-inflammation and auto-antibody-mediated neurological diseases.

## Background

Familial Mediterranean fever (FMF) is a genetic auto-inflammatory disease characterized by self-limiting relapsing fever and polyserositis, caused by mutations of the *MEFV* gene encoding pyrin [1]. Some neurological autoimmune or auto-inflammatory disorders, such as multiple sclerosis, Behçet disease, and polymyositis (PM), have been reported as complications of FMF [2, 3]. We report the first case of Morvan’s syndrome (MoS) and myasthenia gravis (MG) related to FMF harboring *MEFV* mutations (L110P/E148Q).

## Case presentation

**Fig. 1:**
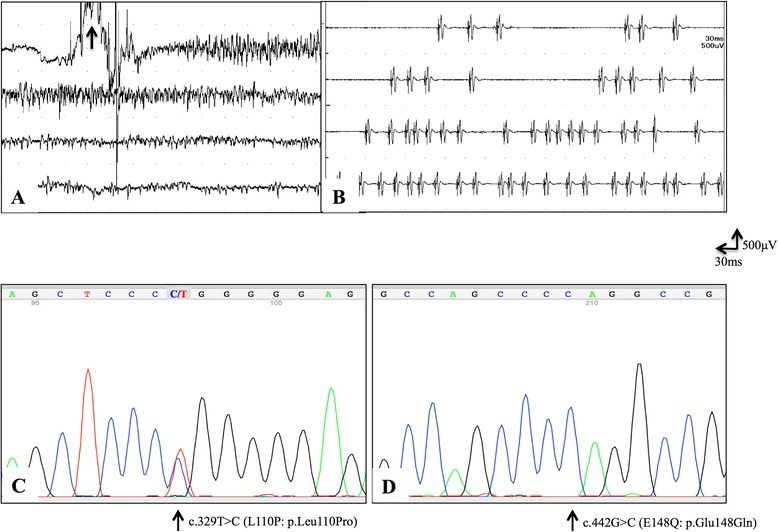
Clinical and pathological findings in the present case. **a**, **b** Needle electromyography at rest shows neuromyotonic discharges around 450 Hz in the left first dorsal interosseous muscle (**a**) and myokymic discharges in the right thenar muscle (**b**). The *black arrow* in **a** indicates an insertional activity. **c**, **d** Analyses of *MEFV* gene mutations identified the heterozygous mutation L110P (**c**) and homozygous mutation E148Q (**d**)

A 40-year-old woman had experienced ptosis and double vision with daily fluctuations for 1 year and was admitted to our hospital in August 2012. She also had insomnia, hyperhidrosis, and progressive weakness of the extremities. She had experienced recurrent fever, arthralgia, abdominal pain, and oral aphtha since childhood; her mother, older sister, and son also had self-limiting periodic fever. Neurologically, she had ptosis, double vision, mild dysphagia, neck flexor weakness (Medical Research Council (MRC) scale grade 4), symmetrical weakness of her deltoids (MRC grades: right 4/left 4), triceps (right 4/left 4), digit flexors (right 3/left 3), quadriceps (right 4/left 4), and tibialis anterior muscles (right 4/left 4), grip myotonia, and myokymia. Myokymia was most frequently seen in the bilateral first dorsal interosseous muscles and less frequently in the lower leg and trunk muscles. An edrophonium test was positive for ptosis, double vision, and proximal limb muscle weakness but not distal muscle weakness. Blood tests were unremarkable except for hyper-IgDemia (12.2 mg/dL; normal, 0.0–9.0). Anti-acetylcholine receptor antibodies were weakly positive (0.4 nmol/L; normal, <0.2) and anti-muscle-specific receptor tyrosine kinase and anti-low density lipoprotein receptor-related protein 4 antibodies were negative. Anti-voltage-gated potassium channel (VGKC) complex antibodies were elevated to 316 pM (normal, <100), while antibodies against leucine-rich glioma inactivated protein 1 (LGI1) and contactin-associated protein-like 2 (Caspr2) were negative. Cerebrospinal fluid tests including IgG index (0.51; normal, <0.73) were normal and oligoclonal IgG bands were absent. Brain and skeletal muscle magnetic resonance imaging and positron emission tomography/computed tomography of the whole body detected no abnormalities including thymic tumors. Needle electromyography showed neuromyotonic discharges in the left quadriceps and first dorsal interosseous muscles (Fig. 1a) and myokymic discharges in the right thenar muscles (Fig. 1b). Nerve conduction studies in the upper and lower extremities were normal. Repetitive nerve stimulation of the orbicularis oculi, frontalis, and abductor digiti minimi muscles showed no decremental or incremental responses. Electroencephalography revealed intermittent irregular slow waves around 4 Hz in the left temporal region without epileptic discharge. Unrelated word pairs (in Miyake’s Paired-Associate Word Learning Test; a Japanese memory function test) elicited low scores: first task = 0 (normal, 3.2–7.0), second task = 4 (normal, 6.6–10.0), and third task = 7 (normal, 7.7–10.0). Based on these observations, we diagnosed her as having MoS complicated with MG. Intravenous methylprednisolone (IVMP) (1 g/day for 3 days) followed by oral prednisolone (PSL) (40 mg/day) with gradual tapering and five courses of simple plasma exchange (PE) alleviated the ptosis, double vision, memory disturbance, hyperhidrosis, and insomnia. The left temporal slow waves were diminished on electroencephalography. The unrelated word pair scores also improved: first task = 3, second task = 9, and third task = 9. However, her myokymia and muscle weakness persisted. Administration of a choline esterase inhibitor (pyridostigmine 180 mg/day) was effective for the ptosis, double vision, and proximal limb weakness but not the distal muscle weakness. Genetic analyses of *MEFV* revealed compound heterozygous mutations of L110P/E148Q in exon 2 (Fig. 1c, d), while analyses for mutations associated with tumor necrosis factor (TNF) receptor-associated periodic syndrome and hyper-IgD syndrome were negative. She had an HLA-B51/-52 genotype. Based on these findings, she was also diagnosed with FMF [1].

Her MG symptoms were exacerbated in January 2013 and she was re-admitted to our hospital. Marked elevation of serum interleukin (IL)-6 (353 pg/mL; normal, <8), TNF-α (43 pg/mL; normal, <2.8), and IL-1β (24 pg/mL; normal, <10) was found. She was treated with IVMP followed by oral PSL (40 mg/day) with gradual tapering, which led to limited efficacy for the ptosis and double vision. Although oral colchicine (1 mg/day) resolved her recurrent fever, arthralgia, and oral aphtha, her MG symptoms frequently recurred from July 2013 to October 2014. PE and IVIg (0.4 g/kg/day for 5 days) were required repeatedly every several months, in addition to PSL (5–10 mg/day) and immunosuppressants (tacrolimus 6 mg/day or azathioprine 100 mg/day), which showed transient efficacy for the ptosis, double vision, and proximal limb weakness. However, the limb weakness with myokymia progressively worsened (right 3/left 3), which rendered her wheelchair-bound.

### Discussion

FMF causes episodic rises in IL-1β and downstream pro-inflammatory cytokines, such as IL-6 and TNF-α, with recurrent fever and serosal inflammation [4]. Indeed, serum IL-1β, TNF-α, and IL-6 were elevated in our patient. Pyrin plays a critical role in assembly of the inflammasome. Although it remains unclear how mutated pyrin produces increases in IL-1β, hyper-activated T helper (Th)1 and Th17 cells are assumed to contribute to the auto-inflammatory response [4].

Interestingly, our patient developed MoS and MG, both antibody-mediated diseases, but her myokymia and MG symptoms were resistant to intensive immunotherapies. A previous report described a case of FMF caused by *MEFV* gene mutations of L110P and E148Q that coexisted with PM [2]. In that case, colchicine treatment successfully alleviated both FMF and PM, suggesting that the disease activity of PM was considerably modulated by the coexisting FMF [2]. Among the pro-inflammatory cytokines elevated in our patient’s serum, IL-6 facilitates Th17 differentiation from naïve T cells and decreases regulatory T cells. Furthermore, pathogenic Th17 cells were shown to exacerbate experimental autoimmune MG [5]. Therefore, frequent pro-inflammatory cytokine surges caused by the *MEFV* mutations, despite their treatment by immunotherapies such as PE, may be responsible for the refractory nature of MG, and possibly MoS, in our case. However, for the development of antibody-mediated neurologic diseases, activation of the adaptive immune system is required, even in patients with non-antigen-specific activation of the innate immune system through a genetic disorder [6].

The reason for the involvement of the distal muscles in our case is unclear. However, one explanation might be that the myokymia and neuromyotonia, which reflect peripheral nerve hyperexcitability (PNH), may play a considerable role because PNH is a potential cause of axon loss that can precede weakness or muscle atrophy [7, 8]. Another explanation could be that the distal muscle weakness was derived from MG because some patients with MG can show distal muscle involvement without other concomitant diseases that contribute to distal muscle weakness [9]; however, a choline esterase inhibitor was only effective for the proximal muscle weakness, and not the distal muscle weakness, in our patient.

## Conclusions

In conclusion, FMF-related auto-inflammation may contribute to the development and augmentation of MG and MoS, suggesting a possible link between auto-inflammation and auto-antibody-mediated diseases.

### Consent to publish

Written informed consent was obtained from the patient for publication of this case report and any accompanying images. A copy of the written consent is available for review by the Editor-in-Chief of this journal. Ethical approval was obtained from the Institutional Review Board of Kagoshima University for anti-VGKC complex antibodies and Kyushu University for *MEFV* gene analyses.

## Abbreviations

Caspr2: contactin-associated protein-like 2
FMF: familial Mediterranean fever
IL: interleukin
IVMP: intravenous methylprednisolone
LGI1: leucine-rich glioma inactivated protein 1
MG: myasthenia gravis
MoS: Morvan’s syndrome
MRC: Medical Research Council
PE: plasma exchange
PM: polymyositis
PNH: peripheral nerve hyperexcitability
PSL: prednisolone
Th: T helper
TNF: tumor necrosis factor
VGKC: voltage-gated potassium channel
